# Constructing a Transient Ischemia Attack Model Utilizing Flexible Spatial Targeting Photothrombosis with Real-Time Blood Flow Imaging Feedback

**DOI:** 10.3390/ijms25147557

**Published:** 2024-07-10

**Authors:** Xuan Zhu, Zichao Yi, Ruolan Li, Chen Wang, Wenting Zhu, Minghui Ma, Jinling Lu, Pengcheng Li

**Affiliations:** 1 Britton Chance Center for Biomedical Photonics and MoE Key Laboratory for Biomedical Photonics, Advanced Biomedical Imaging Facility, Wuhan National Laboratory for Optoelectronics, Huazhong University of Science and Technology, Wuhan 430074, China; zhuxuan@hust.edu.cn (X.Z.); yizichao@hust.edu.cn (Z.Y.); liruolan@hust.edu.cn (R.L.); zhuwenting@hust.edu.cn (W.Z.); maminghui@hust.edu.cn (M.M.); lujinling@hust.edu.cn (J.L.); 2 State Key Laboratory of Digital Medical Engineering, School of Biomedical Engineering, Hainan University, Sanya 572025, China; 3 Research Unit of Multimodal Cross Scale Neural Signal Detection and Imaging, Chinese Academy of Medical Science, HUST-Suzhou Institute for Brainsmatics, Jiangsu Industrial Technology Reserch Institute (JITRI), Suzhou 215100, China

**Keywords:** transient ischemic attack, real-time blood flow imaging, spatial targeting photothrombosis, distal middle cerebral artery, thrombus

## Abstract

Transient ischemic attack (TIA) is an early warning sign of stroke and death, necessitating suitable animal models due to the associated clinical diagnostic challenges. In this study, we developed a TIA model using flexible spatially targeted photothrombosis combined with real-time blood flow imaging feedback. By modulating the excitation light using wavefront technology, we precisely created a square light spot (50 × 250 µm), targeted at the distal middle cerebral artery (dMCA). The use of laser speckle contrast imaging (LSCI) provided real-time feedback on the ischemia, while the excitation light was ceased upon reaching complete occlusion. Our results demonstrated that the photothrombus formed in the dMCA and spontaneously recanalized within 10 min (416.8 ± 96.4 s), with no sensorimotor deficits or infarction 24 h post-TIA. During the acute phase, ischemic spreading depression occurred in the ipsilateral dorsal cortex, leading to more severe ischemia and collateral circulation establishment synchronized with the onset of dMCA narrowing. Post-reperfusion, the thrombi were primarily in the sensorimotor and visual cortex, disappearing within 24 h. The blood flow changes in the dMCA were more indicative of cortical ischemic conditions than diameter changes. Our method successfully establishes a photochemical TIA model based on the dMCA, allowing for the dynamic observation and control of thrombus formation and recanalization and enabling real-time monitoring of the impacts on cerebral blood flow during the acute phase of TIA.

## 1. Introduction

Transient ischemic attack (TIA) is defined as a brief episode of neurological dysfunction caused by focal cerebral, spinal, or retinal ischemia without acute cerebral infarction [1]. Previously considered a benign ischemic event, TIA has been recognized in recent years as an important early warning sign of stroke, with nearly one-third of patients with ischemic stroke (IS) having a previous history of TIAs [2,3]. To improve the diagnostic strategies for TIA and to facilitate the research on TIA-related injury mechanisms, pathological changes, and interventions, it is necessary to establish a suitable animal model that can mimic human TIA and is capable of observing the changes in hemodynamics and neural activity during the onset of TIA.

Most TIA animal models are based on ischemic stroke models and the definition of TIA. The establishment of a successful and reliable TIA model should follow three criteria: (1) objective evidence of cerebral artery occlusion and reperfusion; (2) no permanent neurological deficit; (3) no acute cerebral infarction [4]. The intraluminal suture middle cerebral artery occlusion (MCAO) model is currently the most common method for inducing TIA [5,6]. Wang et al. found that eight minutes of transient MCAO did not result in infarction but led to neuronal apoptosis in the cortex and striatum. They demonstrated that D1-3-N-butylphthalide promotes angiogenesis after TIA by increasing the expression of angiogenic growth factors [7]. Berthet et al. discovered that glutamate could serve as an indicator of recent transient ischemia and that the combined concentration scores of N-acetyl aspartate, glutamate, and taurine are good predictors of irreversible damage as early as three hours after ischemia [8]. The greatest advantage is being able to precisely control the duration of ischemia. However, the infarct thresholds (the minimum durations of ischemia that can cause cerebral infarction) vary in different animals. The distal MCAO model, in which the blood flow of the distal middle cerebral artery (dMCA) is temporarily interrupted using a microvascular clip [9] or microsurgical hook lift [10], similarly allows the ischemia duration to be controlled. Research has shown that although there is no substantial brain damage after a TIA, there is still selective neuronal loss in the cortex. Additionally, microglial activation may serve as a new therapeutic target. The main advantage of using this method to establish a TIA model is seen in the consistent infarct thresholds observed across different mouse and rat strains. Nonetheless, the rapid and complete recanalization of blood vessels induced by both models does not align with the clinical presentation of TIA. Moreover, these two models are ineffective for anticoagulant and antiplatelet treatments.

The photothrombosis model uses photosensitive agents (such as Rose Bengal (RB) dye) to generate reactive oxygen species (ROS) through photo-oxidation. This process causes endothelial damage, leading to platelet aggregation and thrombus formation under the influence of tissue factor (TF), ultimately resulting in vascular occlusion and ischemia [11]. The mechanism of photothrombotic injury closely mirrors the fundamental processes of human TIA, including the basic inflammatory response [12]. Compared to other methods, this model, particularly when linked to thrombin, facilitates intervention and treatment experiments using clinically common thrombolytic and anticoagulant drugs such as tissue plasminogen activator (tPA) and aspirin [13]. Additionally, the photothrombosis model offers the advantage of precisely controlling the infarction area and location by adjusting the irradiation beam. The extent of induced ischemia can be regulated by varying the light dosage, leading to high reproducibility and a higher survival rate [14]. Therefore, the photothrombotic model serves as an important tool for studying therapies related to TIA.

There are two main approaches to establish a photochemical TIA model. The traditional approach is using a low-intensity laser beam with a bright center, a low dose of Rose Bengal photosensitizer, and a short five to fifteen minute illumination period [15,16]. Nonetheless, these photothrombosis models still have tiny infarcts in the cortex at twenty-four hours, which does not correspond with the absence of cortical infraction in clinical TIA. Another alternative is the TIA model based on photothrombosis of the small vessels of the rodent brain. Using two-photon technology, it is possible to temporarily block the blood flow by irradiating single arteries, such as pial vessels [17], capillaries [18], and penetrating arteries [19]. However, because these models embolize the terminal arteries directly, they affect the establishment of collateral circulation and also cannot be used to observe the physiological changes during the acute phase of TIA. Simultaneously, these experimental methods are challenging for TIA establishment based on large cerebral vessels, such as the middle cerebral artery, which is one of the primary sites of ischemic stroke in humans. However, in MCA-targeted photochemical ischemia studies, illumination tools such as mirrors [20] and optical fibers [21] have been used. These methods produce relatively large light spots, covering the entire distal MCA (dMCA) or larger areas, resulting in cerebral infarction but failing to induce transient ischemic attacks (TIAs). Therefore, we aimed to establish a photothrombotic TIA model based on the MCA that can accurately simulate and monitor the acute phase of TIA without causing acute cerebral infarction.

Laser speckle contrast imaging (LSCI) velocity information was obtained by calculating the contrast of speckles formed by the scattering of moving cells and molecules within blood vessels [22]. LSCI has advantages such as its non-contact operation, wide field of view, and high resolution, providing an effective wide-field imaging method for real-time blood flow imaging [23]. Spatial light modulators (SLMs) are phase-type wavefront modulation devices that have been widely used in recent years. Using computational holography, the phase of the stimulating light is modulated to generate various diffraction patterns, enabling light irradiation at different positions and in different shapes [24].

In this study, we used SLMs to modulate the excitation light, achieving flexible spatially targeted photothrombosis. The excitation light was shaped into a square spot with a width of approximately half the diameter of the dMCA (50 × 250 µm). The LSCI provided real-time blood flow imaging feedback during ischemia to precisely control the cessation of illumination for each mouse upon reaching complete occlusion. This approach facilitated the formation of an unstable photothrombus in the dMCA and enabled spontaneous recanalization, thereby establishing a model of photochemical transient cerebral ischemia in the dMCA. By integrating blood flow imaging of the dorsal cerebral cortex (dCC), we dynamically observed in real time the entire process of photothrombotic ischemia in the distal middle cerebral artery region, along with the spatiotemporal dynamics of dorsal cerebral cortex blood flow, including the collateral circulation, throughout the acute phase of a TIA. In addition, the injection of antibody-labeled platelets in vivo allowed the identification of thrombus distribution in the cortex post-reperfusion.

## 2. Results

### 2.1. Establishment of a Photochemical TIA Model

#### 2.1.1. Condition 1: Occlusion and Reperfusion of the Distal Middle Cerebral Artery

**Figure 1:**
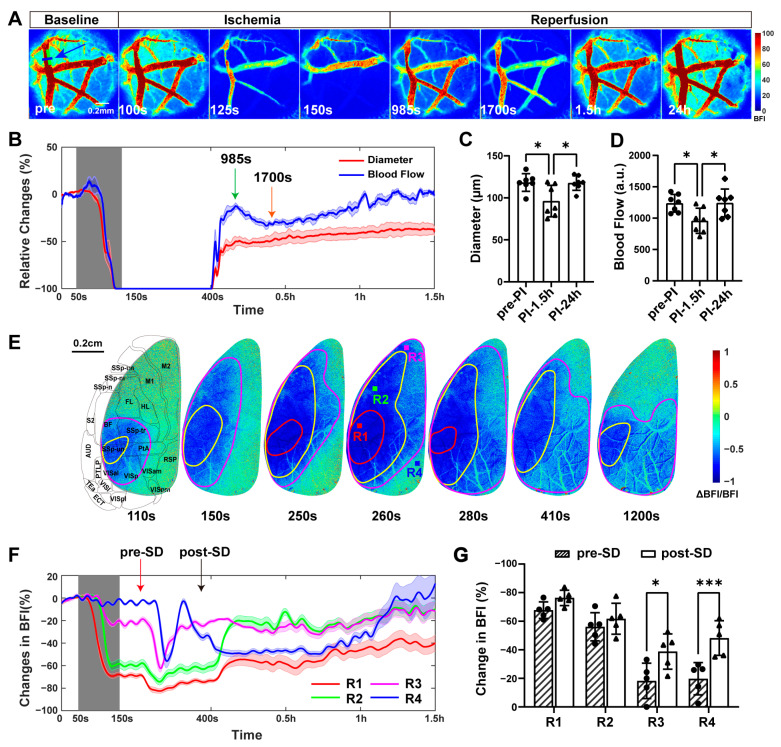
Representative blood flow changes during the TIA. (**A**) Representative BFI images of the dMCA at various time points during baseline, ischemia, and reperfusion. The green box in the baseline image indicates the position of the light spot, with the dMCA blood flow direction from top to bottom. (**B**) Relative changes in diameter (red) and blood flow (blue) for one representative mouse over time at the blue line indicated by a blue arrow in the baseline image (**A**), with the gray area indicating the duration of light. Green arrow points to 985 s and orange arrow points to 1700 s. (**C**) The diameter of the dMCA in the light spot for different mice pre-PI, at 1.5 h post-PI, and at 24 h post-PI (*n* = 7). Note: *p* value based on one-way repeated-measures ANOVA followed by a post hoc Tukey’s test. (**D**) The blood flow of the dMCA in the light spot for different mice pre-PI, at 1.5 h post-PI, and at 24 h post-PI (*n* = 7). Note: *p* value based on one-way repeated-measures ANOVA followed by a post hoc Tukey’s test. (**E**) Representative relative change BFI images of the left hemisphere in the dCC. The diagram on the first image illustrates the corresponding structural brain regions. Areas outlined in magenta indicate blood flow reduction to 80% of baseline or less, those in yellow indicate 40% or less, and those in red indicate 20% or less. BF: barrel field; SSp: primary somatosensory cortex; FL: forelimb; HL: hindlimb; M1: primary motor area; M2: secondary motor area; VISp: primary visual area; RSP: retrosplenial area. (**F**) Relative blood flow changes over time for regions R1-R4 in (**E**) for one mouse. Red arrow points to the time pre-SD and black arrow points to the time post-SD. (**G**) Relative BFI changes of regions of interest R1-R4 (**E**) pre- and post-SD. Note: *p* values based on paired *t*-test (*n* = 5); * *p* < 0.05, *** *p* < 0.001.

A cerebral ischemia system that utilized real-time blood flow imaging feedback and flexible spatially targeted photochemistry generated a light spot on the upstream of the dMCA that was approximately half the width of the vessel’s diameter and five times as long as its width (50 × 250 μm; green box in the baseline image of Figure 1A). The illumination was halted after reaching complete occlusion, enabling the thrombi to spontaneously recanalize within ten minutes (416.8 ± 96.4 s). The use of LSCI at different wavelengths enabled the simultaneous and real-time monitoring of hemodynamic changes within the dMCA and the dorsal cerebral cortex during the acute phase of PI. Through the blood flow index (BFI) images, the gradual narrowing to complete occlusion followed by the reperfusion of the dMCA was observable, along with the ischemia and reperfusion in the dorsal cortex (Figure 1). Figure 1A demonstrates the changes in dMCA window BFI images over time. At the onset of reperfusion, there was an increase in dMCA blood flow (985 s; indicated by green arrow in Figure 1B), followed by a slight decrease (1700 s; indicated by orange arrow in Figure 1B) and then a slow and gradual recovery. Significant differences in dMCA diameter (Figure 1C) and blood flow (Figure 1D) were observed between the pre-PI period and 1.5 h post-PI, as well as between 1.5 h and 24 h post-PI. These findings suggested ischemia and subsequent reperfusion of the dMCA among seven mice.

The ischemia was initiated in the barrel field (BF) and primary somatosensory cortex (SSp), followed by ischemia in the forelimb (FL) and hindlimb (HL) areas, spreading to the primary (M1) and secondary motor (M2) cortexes and subsequently extending to the retrosplenial (RSP) and visual (VISp) cortexes (Figure 1E). Brain tissue with BFI changes within 20% of the baseline were considered to have normal perfusion. Following cerebral ischemia, the brain tissue were categorized into different blood perfusion environments based on the degree of local blood supply, resulting in three distinct scenarios. A decrease in BFI to 40%–80% of the baseline was classified as benign ischemia (area between the magenta and yellow outlines in Figure 1E), a reduction to 20%–40% of the baseline was defined as the ischemic penumbra (areas between the red and yellow outlines in Figure 1E), and a drop to less than 20% of the baseline was identified as an infarction (areas outlined in red in Figure 1E) [25,26]. Between the red arrow and the black arrow in Figure 1F, the regions of interest (ROIs; R1–R4) reached their troughs sequentially, followed by a gradual recovery in BFI. The results indicated that during the phase of occlusion of the dMCA, ischemic spreading depression (SD) occurred. Before and after the SD, there were significant reductions in BFI in regions R3 and R4 (Figure 1G). The SD led to ischemia in areas supplied by arteries other than the middle cerebral artery. However, the extent of ischemia in these areas was significantly less than in the regions directly supplied by the middle cerebral artery. We observed that during the complete occlusion phase of PI, either no SD occurred or only a single SD took place, with a 71.4% probability of the latter, which was different from the multiple waves of SD that occurred during permanent ischemia [27].

#### 2.1.2. Conditions 2 and 3: No Permanent Neurological Deficits and No Acute Cerebral Infarction

**Figure 2:**
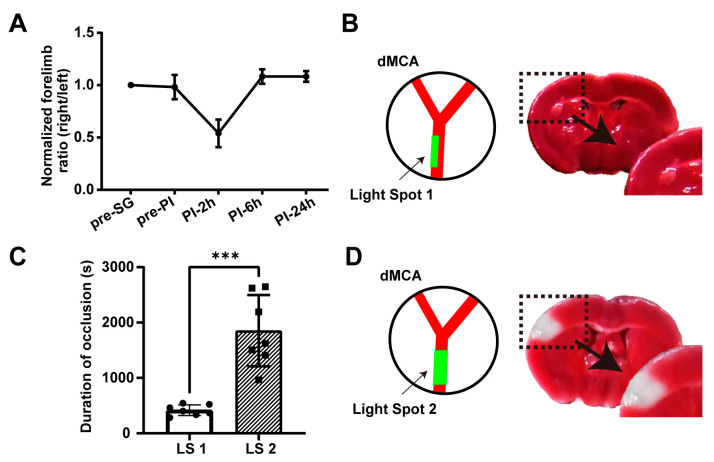
Behavioral and histological staining. (**A**) Cylinder test results at various time points before and after PI with light spot 1 (*n* = 7). (**B**) Representative coronal brain sections of TTC staining within 24 h post-PI with light spot 1. Lower right corner shows a magnified view of the black dashed box. (**C**) Durations of occlusion in different light spots. LS 1: green box in the left panel of (**B**) (*n* = 7); LS 2: green box in the left panel of (**D**) (*n* = 7). Note: *p* value based on unpaired *t*-test, *** *p* < 0.001. (**D**) Representative coronal brain sections of TTC staining within 24 h post-PI with light spot 2. Lower right corner shows a magnified view of the black dashed box, while the white area indicates the infarct.

In the above, we successfully induced ischemia–reperfusion in the dMCA. The cylinder test was employed to assess the behavioral changes in these mice (*n* = 7) at various time points, namely before surgery (pre-SG); before PI (pre-PI); and at 2 (PI-2h), 6 (PI-6h), and 24 (PI-24h) hours post-PI (Figure 2A). The baseline was established by the ratio of wall touches by the left and right limbs before surgery and normalized by this baseline. No significant differences were observed in the behavior of mice before and after surgery nor between the pre-ischemic state and the state 24 h post-ischemia, indicating the absence of neurological deficits. The brain tissue samples underwent 2,3,5-triphenyl tetrazolium chloride (TTC) staining 24 h post-PI. The outcomes revealed that all brain tissue samples were stained red without any white ischemic lesions, suggesting no acute infarction in the ischemic model at 24 h (Figure 2B). Thus, we confirmed that the model met the three criteria for establishing a TIA model. This demonstrated that we had successfully established a dMCA-based photothrombotic TIA mouse model. Additionally, we used a light spot with a width equal to the vessel width (light spot 2 (LS 2) in Figure 2D) to simulate traditional large-spot illumination, while maintaining the light power density consistent with that of LS 1, and stopped the illumination upon complete occlusion. We found that the duration of occlusion with LS 2 was significantly longer than with LS 1 (1852 ± 644 s vs. 416.8 ± 96.4 s, *p* < 0.001; Figure 2C). Moreover, the TTC staining 24 h post-PI with LS 2 showed obvious infarction at the dMCA site (Figure 2D). This proved that controlling the light spot size was crucial for establishing the TIA model.

### 2.2. Establishment of Collateral Circulation Observed in the Dorsal Cortex during TIA

**Figure 3:**
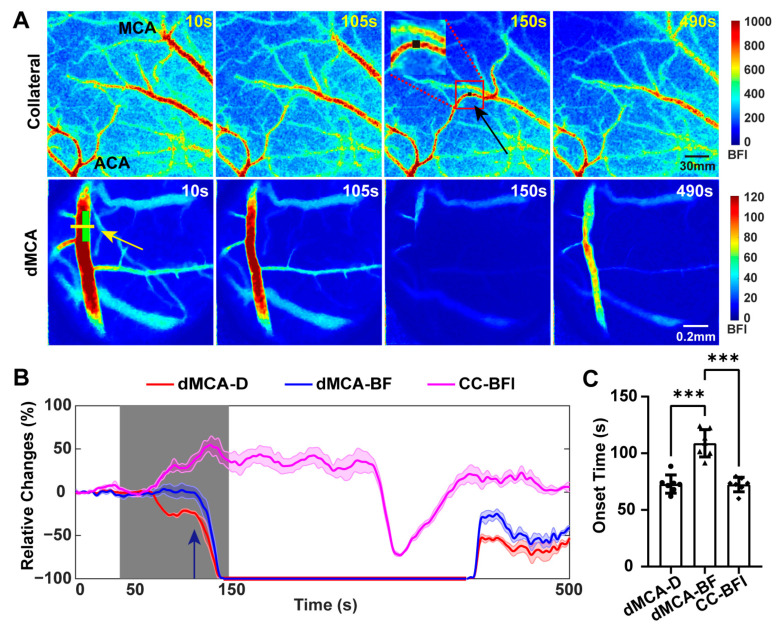
Establishment of collateral circulation during the TIA. (**A**) Representative blood flow images of collateral circulation in the dorsal cortical cortex (top row) and dMCA (bottom row) at different time points. In the third image, the black arrow points to the collateral circulation and the black square in the enlarged view within the red box indicates the ROI selected for the collateral circulation. The green box in the first image of the bottom row indicates the position of the light spot. (**B**) Typical relative changes over time of diameter (dMCA-D; red) and blood flow (dMCA-BF; blue) at the yellow line indicated by the yellow arrow in (**A**) and relative changes in blood flow index of the collateral circulation in the ROI selected in (**A**) over time (CC-BFI, magenta) for one representative mouse. The gray area indicates the green light exposure time and the dark blue arrow points to the moment when the blood flow of the dMCA began to decrease. (**C**) The bar graph illustrates the onset times of change for different mice (*n* = 7), showing the onset of narrowing in the dMCA diameter, the beginning of a reduction in dMCA blood flow, and the initial increase in blood flow index within the collateral circulation. Note: *p* value based on one-way repeated-measures ANOVA followed by a post hoc Tukey’s test, *** *p* < 0.001.

The traditional photochemical ischemia produced consistent infarcts within the cortex [28]. The lesion induced by this method was resistant to therapies based on the enhancement of collateral perfusion because of its terminal arterial occlusive nature [29]. The photochemical ischemia based on the dMCA did not affect the establishment of collateral circulation in the dorsal cerebral cortex. The representative real-time blood flow imaging of the dorsal cerebral cortex enabled the monitoring of changes in collateral circulation during the acute phase of a TIA (Figure 3A). The blood flow transitioned from the anterior cerebral artery (ACA) towards the middle cerebral artery. The collateral circulation was rapidly established during progressive occlusion of the dMCA and gradually weakened after reperfusion of the dMCA. Following the initiation of illumination, the vascular diameter at the dMCA light spot site began to decrease and the BFI index of the collateral circulation started to rise. At the time indicated by the dark blue arrow in Figure 3B, the diameter of the dMCA decreased, while the blood flow remained constant at the baseline level. At this point, the BFI of the collateral circulation had already increased to some extent. As the diameter of the dMCA further narrowed and the blood flow began to decrease, the BFI of the collateral circulation correspondingly increased. The time for seven mice at which the collateral circulation blood flow began to increase (72.4 ± 6.4 s) was close to the time of onset of narrowing of the diameter of the dMCA (72.9 ± 8.1 s), although both were significantly earlier than the time (108.9 ± 6.4 s) at which the blood flow through the dMCA started to decrease (Figure 3C, *n* = 7). When the diameter of the dMCA initially started to narrow, downstream vessels started to constrict. At this stage, even though the blood flow in the dMCA remained at baseline levels, it led to the establishment of collateral circulation.

### 2.3. Thrombus Could Be Observed Downstream from the dMCA after TIA

**Figure 4:**
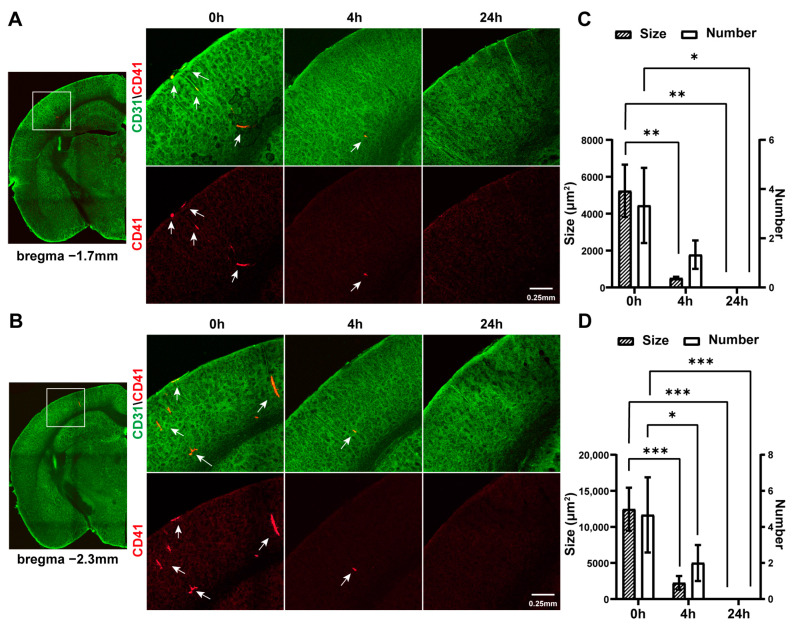
The representative distribution of cortical thrombi at different times after transient ischemia. Brain slices at bregma −1.7 mm (**A**) and bregma −2.3 mm (**B**). The images on the right are enlarged views of the areas within the white boxes in the images on the left at 0 h, 4 h, and 24 h: (**A**) sensorimotor cortex; (**B**) visual cortex). White arrows indicate the locations of thrombi. Green: CD31, labelled endothelial cells; red/orange: CD41, labelled platelets. (**C**) The area and number of thrombi in the sensorimotor cortex at different times after transient ischemia (*n* = 3). Note: *p* value based on one-way ANOVA followed by a post hoc Tukey’s test. (**D**) The area and number of thrombi in the visual cortex at different times after transient ischemia (*n* = 3). Note: *p* value based on one-way ANOVA followed by a post hoc Tukey’s test; * *p* < 0.05, ** *p* < 0.01, *** *p* < 0.001.

The photothrombus formed in the dMCA and spontaneously dissolved within ten minutes, and reperfusion was subsequently observed. The thrombus separated from the dMCA and followed the direction of blood flow moving to the terminal cortical artery and then to the subcortical perforating artery, thereby occluding the downstream vessels. In order to track the thrombus’ location, platelets and endothelial cells were labeled with specific antibodies. Subsequently, the brains were carefully removed at 10 min (as 0 h), 4 h, and 24 h after ischemia–reperfusion for sectioning and imaging (Figure 4). Immediately after ischemic recanalization (0 h), significant platelet aggregation and blood clots (indicated by white arrows) were observed in the sensory cortex and visual cortex (indicated by white boxes in Figure 4, left panels), with the thrombi separated from the dMCA obstructing the penetrating arteries. At 4 h post-ischemia, the thrombi were noticeably reduced. At 24 h post-ischemia, no significant thrombi were observed. This indicated that after transient ischemia in the dMCA, the thrombi were primarily concentrated in the sensorimotor and visual cortexes, and their size (12,458.7 ± 2988.7 µm^2^ (0 h) and 2239.4 ± 950.8 µm^2^ (4 h), for example, in Figure 4D) and number (4.67 ± 2.08 (0 h) and 2 ± 1 (4 h), for example, in Figure 4D) gradually decreased over time, eventually disappearing after 24 h (Figure 4C,D).

### 2.4. Blood Flow in the dMCA Acted as a More Reliable Indicator of Ischemic Changes in the Cortex Than the Vascular Diameter Did

Based on the blood flow changes associated with dMCA ischemia, the entire acute phase of the TIA was divided into four phases. The phase during which the blood flow remained essentially unchanged, before and for a short period after light exposure, was defined as the baseline phase. The phase where the blood flow or diameter began to decrease following complete occlusion was defined as the stenosis phase. The phase characterized by a reduction in blood flow to less than 10% of the baseline level was defined as the complete occlusion phase, during which the vessel diameter was considered to be zero. The stage following the onset of blood flow recovery was defined as the reperfusion phase.

**Figure 5:**
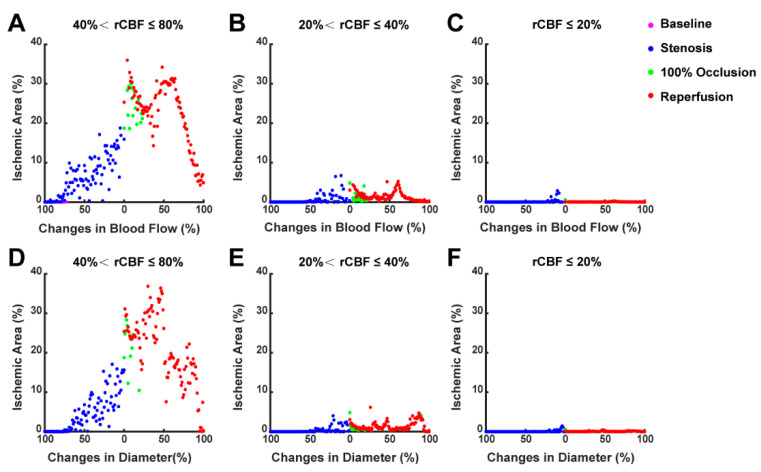
The relationship of both the diameter and blood flow of the dMCA with the ischemic area in the dorsal cortex during the acute phase of the TIA (*n* = 7): (**A**–**C**) the relationship between the changes in dMCA blood flow and the ischemic area in the dorsal cortex; (**D**–**F**) the relationship between the changes in dMCA diameter and the ischemic area in the dorsal cortex. Benign ischemia: 40% < rCBF ≤ 80% (**A**,**D**); penumbra: 20% < rCBF ≤ 40% (**B**,**E**); infarction: rCBF ≤ 20% (**C**,**F**). Magenta dots indicate the baseline phase, blue dots indicate the stenosis phase, green dots indicate the 100% occlusion phase, and red dots indicate the reperfusion phase.

The horizontal axis in Figure 5 represents the changes in blood flow (Figure 5A–C) and diameter (Figure 5D–F) in the dMCA light spot among seven mice. The left half of each image represents the gradual narrowing from baseline to complete occlusion (100% to 0%), while the right half depicts the gradual recovery after reperfusion (0% to 100%). The vertical axis indicates the proportion of the ischemic region’s area in relation to the overall area of the dorsal cortex, across different levels of ischemia. Overall, the cases of cortical ischemia were predominantly benign, with the maximum proportion of the benign ischemic area reaching 38%. The proportions of the penumbral area and infarct area were below 8% and 3%, respectively. The duration of the infarct region lasted for less than one minute, which was insufficient to form an acute infarction. The red dots were located higher than the blue dots at the same degree of change (e.g., stenosis to 50% of baseline vs. reperfusion recovery to 50% of baseline in Figure 5A,B), indicating that when the dMCA was at the same degree of ischemia, the ischemic area of the dorsal cortex in the reperfusion phase was larger than in the stenosis phase. The changes in diameter and blood flow at the dMCA were generally linearly correlated with the cortical ischemic area, whereby the greater the degree of stenosis, the larger the area of cortical ischemia; conversely, the more extensive the reperfusion recovery, the more reduced the ischemic area.

The magenta dots indicate the baseline phase, which partially overlap with the blue dots. During the stenosis phase (blue dots), when the diameter and blood flow of the dMCA decreased to approximately 75% and 85% of the baseline, respectively, benign ischemia began to appear in the cortex. When both the diameter and blood flow reached around 50% of the baseline, the ischemic penumbra emerged in the cortex. The green dots indicate the stage of 100% occlusion, during which the ischemic area gradually increased. During the reperfusion phase (red dots), the relationship between the blood flow and cortical ischemic area displayed an inverse “N” shape, characterized by a triphasic change, with a trough at 25% and a peak at 60% (Figure 5A). When the photothrombus was just separated from dMCA, there was a rapid increase in blood flow in the terminal branches of the MCA in the dorsal cortex, significantly alleviating the ischemia and reducing the ischemic area. Subsequently, the dMCA remained in a state of ischemia. The area that initially experienced a rapid increase in blood flow underwent a reduction in blood flow once again, leading to a reverse expansion of the ischemic area. When the blood flow was restored to 60% and above, the cortical ischemia was gradually alleviated and the ischemic area progressively decreased. Similarly, for the diameter, there was also a peak (at 35%) and a trough (at 20%), although the trend of the changes was not as pronounced as that observed for the blood flow (Figure 5D). The scatter plot of the diameter versus ischemic area was more dispersed compared to the blood flow, indicating that the blood flow in the dMCA was a more reliable indicator of cortical ischemic changes than the vascular diameter.

**Figure 6:**
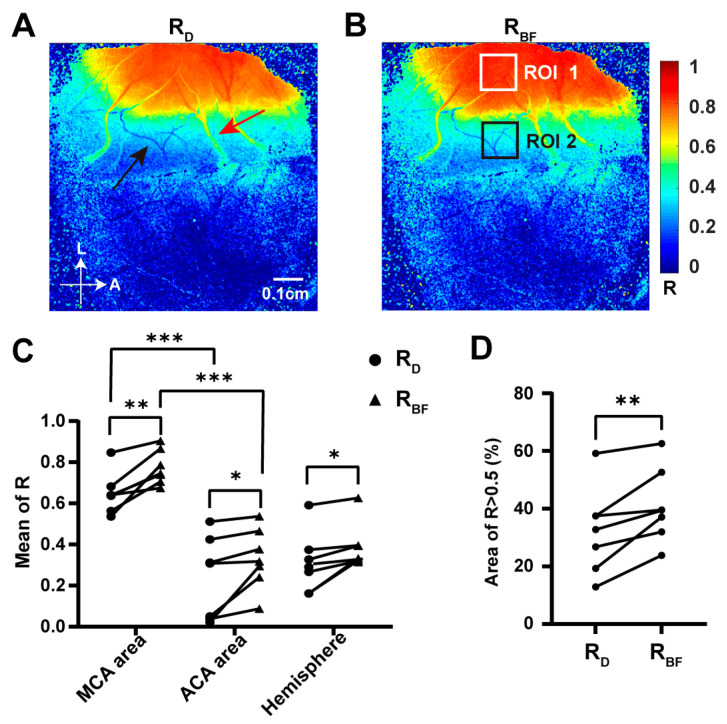
The relationship of both the diameter and blood flow of the dMCA with the dorsal cortical blood flow during the acute phase of the TIA. (**A**) Representative distribution map of correlation coefficients (RD) between relative changes over time in the diameter of the dMCA and BFI across each pixel of the dCC, with the ACA indicated by a black arrow and cortical veins indicated by red arrows. (**B**) Representative distribution map of correlation coefficients (RBF) between relative changes over time in blood flow of the dMCA and BFI across each pixel of the dCC, MCA area (ROI 1; white box), and ACA area (ROI 2; black box) (**C**) Mean values of the RD and RBF for the MCA area, ACA area, and entire left hemisphere (*n* = 7). Note: *p* value based on paired *t*-test. (**D**) Proportions of certain areas (RBF> 0.5 and RD > 0.5) relative to the entire left hemisphere (*n* = 7). Note: *p* value based on paired *t*-test; * *p* < 0.05, ** *p* < 0.01, *** *p* < 0.001.

The relationship between changes in the dMCA and blood flow in the dorsal cortical is reflected by the Pearson correlation coefficients for the relative changes in diameter (R_D_) and blood flow (R_BF_) at the dMCA light spot with the relative changes over time in BFI across each pixel of the dCC. Representative distribution maps of R_D_ and R_BF_ are presented in Figure 6A,B. The correlation was particularly strong (R > 0.5) in areas directly supplied by the MCA and in the regions of the cortical veins (indicated by a red arrow in Figure 6A). Additionally, it was found that the R_D_ and R_BF_ in the cortical ACA (marked by a black arrow in Figure 6A) were lower compared to the adjacent areas, suggesting asynchronous changes in the ACA owing to the establishment of collateral circulation. The mean values of the R_D_ and R_BF_ in the MCA-supplied areas were significantly higher than those in the ACA-supplied areas (Figure 6C, *p* < 0.001), indicating a stronger correlation between the diameter or blood flow of the dMCA and the cortical BFI in regions supplied by the MCA. Moreover, the mean values of R_BF_ in the MCA area (ROI 1; white box in Figure 6B), the ACA area (ROI 2; black box in Figure 6B), and the entire left hemisphere were significantly greater than the mean values of R_D_, suggesting a more pronounced correlation strength between the blood flow of the dMCA and dorsal cortical BFI compared to that of the diameter (0.77 ± 0.08 vs. 0.65 ± 0.1, 0.33 ± 0.15 vs. 0.24 ± 0.2, 0.38 ± 0.11 vs. 0.31 ± 0.15, all *p* < 0.05; Figure 6C). Regions with a correlation coefficient greater than 0.5 were characterized as strongly correlated areas. The area where R_BF_ > 0.5 was significantly larger than the area where R_D_ > 0.5 (41 ± 13% vs. 32 ± 15%, Figure 6D) indicated that the region of strong correlation between the blood flow of the dMCA and cortical blood flow was more extensive than that for the diameter. Thus, both the mean correlation strength and range of strong correlations between the blood flow at the dMCA and cortical blood flow were significantly higher than those observed between the diameter at the dMCA and cortical blood flow. These findings further underscore the notion that the blood flow more accurately reflects changes in cortical blood flow.

### 2.5. Inducing Secondary Transient Ischemia at the Same Location in the dMCA

**Figure 7:**
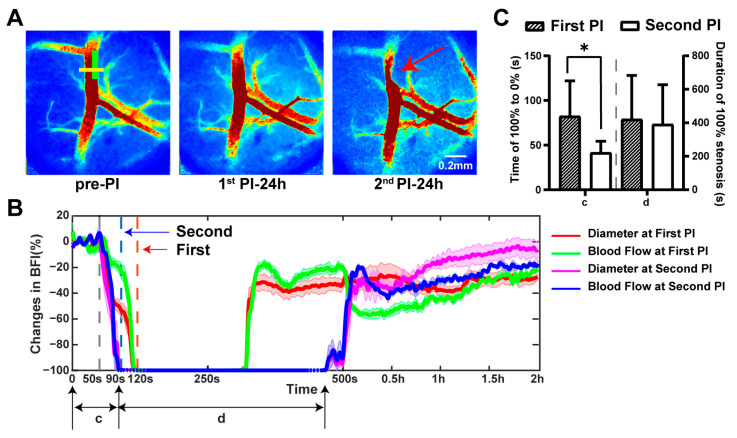
Changes in blood flow during two transient ischemia events. (**A**) Representative BFI images at three time points: before photochemical ischemia (pre-PI); 24 h after first photochemical ischemia (1st PI-24h); 24 h after second photochemical ischemia (2nd PI-24h). The green box indicates the position of the light spot. Red arrow indicates the stenosis position. (**B**) Representative relative changes in diameter (red: first PI; magenta: second PI) and blood flow (green: first PI; blue: second PI) at the yellow line in (**A**) for one representative mouse over time. The grey dotted line marks the onset of light exposure, the red arrow indicates the cessation of the first ischemic light exposure, and the blue arrow indicates the cessation of the second ischemic light exposure. Here, c indicates the time from the start of light to complete occlusion and d indicates the duration of complete occlusion. (**C**) This panel shows c and d at the time of the first PI and second PI (*n* = 6). Note: *p* value based on paired *t*-test; * *p* < 0.05.

TIAs often occur more than once, with one-third of TIA patients experiencing recurrent episodes [30]. Compared to a single event, recurrent TIAs, frequently observed within one day of the initial episode, significantly increase the risk of a severe stroke [31]. We provided an approach that allowed us to precisely generate a secondary ischemia at the same position (Figure 7). Twenty-four hours after inducing the first TIA (first PI), a second PI was induced in the same manner at the same location on the dMCA (second PI). As shown in the third image of Figure 7A, stenosis persisted at the dMCA illumination site following the second ischemic event (red arrow in Figure 7A). This suggested that the thrombus formed during the second PI was more stable, with persistent stenosis of the dMCA for over 24 h in 2 out of 6 mice. We observed that the time from the onset of illumination to complete occlusion during the second PI (41.5 ± 12.8 s) was significantly shorter than during the first PI (82.3 ± 39.6 s), indicating a faster rate of ischemia. However, the durations of complete occlusion showed no significant difference (Figure 7B,C).

## 3. Discussion

To improve the diagnostic strategy for TIA, it is necessary to establish a suitable animal model that can mimic human TIA. In this study, by employing flexible spatial targeting photochemistry and real-time blood flow imaging feedback, the photochemical TIA model without obvious infarction was successfully established, which aligns more closely with the clinical definition.

The methods used for modeling TIA in previous studies still have some limitations. TIA models constructed with filament MCAO and distal MCAO are inconsistent with clinical TIA in terms of rapid and complete revascularization and are ineffective for coagulation and antiplatelet therapy [32]. In contrast, constructing a TIA model using photothrombosis can effectively overcome these issues. Additionally, this method allows for the induction of ischemic damage in specific brain regions through coupling with optical systems and ensures high reproducibility. Previous MCA-based photochemical ischemia models used mirrors and optical fibers, which produced large light spots, making it difficult to avoid brain infarction [33]. Although the presence of large infarcts was avoided by reducing the light dose and RB concentration, small infarct foci still existed, which is inconsistent with the clinical definition of TIA [34]. While methods combining optical techniques such as two-photon microscopy [35,36] can temporarily block the blood flow in small cortical vessels without causing acute infarcts, they induce terminal artery ischemia, affecting the establishment of collateral circulation. Additionally, the use of photothrombosis based on two-photon technology makes it difficult to achieve transient ischemia in large vessels such as the MCA, which is one of the most commonly affected vessels in ischemic stroke in humans. In our study, we used wavefront modulation technology to flexibly control the shape and position of light spots for spatially targeted photothrombosis, coupled with real-time blood flow imaging feedback from the LSCI, allowing for precise control of the light dose. Unlike traditional large light spots that may cause acute infarction, we precisely controlled a light spot with a width that was half the dMCA diameter and forced the illumination to stop upon complete occlusion in each mouse by employing this method. This successfully established a dMCA-based photothrombotic TIA mouse model.

Additionally, our method allows for real-time blood flow imaging of the dMCA and dCC, providing insights into the hemodynamic changes during the acute phase of TIA. We found that the blood flow in the dMCA was a more reliable indicator of ischemic changes in the cortex than the vascular diameter. In a previous study, acute ischemic stroke patients with MCA stenosis, especially those with combined extracranial ICA stenosis, had more severe neurological deficits and worse outcomes [37]. The clinical attention was particularly focused on the degree of stenosis; however, our results suggested that blood flow, as an indicator, should receive more attention. Ischemic spreading depression occurred during the complete occlusion of the dMCA and did not occur after reperfusion. This correlates with clinical symptoms such as dysphasia, amaurosis fugax, and hemiparesis. Decreased nitric oxide (NO) levels and increased potassium ion (K+) concentrations cause synergistically enhanced vasoconstriction and inhibited vasodilation, leading to spreading of the depolarization [38]. This results in further vessel constriction, a severe reduction in cerebral blood flow, and ultimately spreading of the ischemia. The propagation of ischemic SD affects neurovascular coupling and the establishment of collateral circulation, expanding the cortical penumbra, which may further induce apoptosis and necrosis [14]. Additionally, ischemic SD causes microvascular dysfunction and blood–brain barrier disruption, promoting edema and inflammation, which contribute to secondary brain damage [39]. In our model, the observations of ischemic spreading depolarization (SD) suggest that the depolarization wave could be a potential therapeutic target to mitigate secondary injury and reduce the incidence of subsequent strokes. When blood flow was restored to the ischemic brain tissue, the oxidative stress and DNA damage increased, exacerbating the endothelial inflammation. This inflammation manifested as the release of cytokines and the recruitment of immune cells, directly or indirectly increasing the extent of brain injury and neurological deficits [40]. Although the transient ischemia did not cause acute cerebral infarction, it could lead to selective neuronal necrosis and microglial activation [9,41], which might also be associated with ischemia–reperfusion injury. Our model precisely simulated the processes of ischemia and reperfusion, providing a controlled platform for studying the complex relationship between hemodynamics and neuronal injury. This was crucial for understanding the pathological mechanisms of TIA. Additionally, our model allowed for real-time observations of thrombosis and recanalization, enabling dynamic monitoring of the therapeutic effects of anticoagulant and antiplatelet drugs during the acute phase. This approach is invaluable for optimizing treatment strategies and dosages.

TIA usually occurs more than once, and recurrent TIA leads to a higher risk of severe stroke compared to a single TIA [30,42]. However, surprisingly little is known of the interactions of the ischemic injury processes that occur with multiple mild ischemic insults. Wang et al. established a recurrent TIA model involving three 10 min periods of middle cerebral artery occlusion within a week in rats, evaluating the recurrent TIA-induced neuropathological changes [43]. Tuor et al. reproduced mild cerebral ischemia in the MCA using a microclip, discovering that early recurrence caused greater damage than subacute recurrence [44]. In our research, through the use of flexible spatial targeting photothrombosis and real-time blood flow imaging feedback, we could precisely induce a secondary transient ischemia in the same position. During the acute phase of TIA, the rate of second ischemia was significantly faster than that of the first event. This indicated that during the secondary ischemic events, platelets, influenced by integrins and other molecules [45], formed thrombi more rapidly by binding with fibrin. Additionally, the thrombus formed by the second ischemia might lead to persistent stenosis of the dMCA lasting beyond 24 h, suggesting that the thrombus was more stable. These results might suggest that acute secondary ischemia may lead to more severe ischemic damage. According to Stalker’s study, tightly packed thrombus cores contained large amounts of fibrin and were structurally stable, whereas the loosely packed outer shell contained less fibrin and was more easily disrupted by blood flow [46]. Therefore, to further study the differences in thrombus formation between the two ischemic events, co-labeling of the platelets and fibrin could be used in the future. This approach would allow for a more in-depth understanding of the pathological mechanisms of thrombus formation in secondary ischemia by observing the interactions between fibrin and platelets.

Multiple ministrokes are often found in elderly patients with cognitive impairment, vascular dementia, or dementia associated with Alzheimer’s disease [47]. Embolic ministroke models, which include the administration of cholesterol crystals [48], lipid microparticles [49], or natural clots [50], serve as methods to simulate these conditions. Unfortunately, vascular photothrombosis did not adequately model the occurrence of multiple microinfarcts. With our system, it is possible to simultaneously target multiple locations for ischemia, which may lead to multiple ministrokes. Additionally, with our TIA model, thrombi are observed in the sensorimotor and visual cortex after the thrombus is separated from the dMCA. This is due to the fact that the terminal branches of the dMCA in the dorsal cortex are mainly distributed in these areas [51]. By manipulating the light dose, we were able to intermittently induce the formation of unstable thrombi within the distal middle artery. This process may result in the generation and subsequent dislodgement of numerous microthrombi, which could then migrate and occlude downstream vessels. Therefore, our method provides a possible strategy for inducing multiple ministrokes via photothrombosis.

In our study, mice were anesthetized using isoflurane, which competitively inhibits NMDARs at the glycine site [52]. This inhibition may reduce the likelihood of infarction at the irradiation site, thereby facilitating the establishment of our TIA model. Additionally, anesthesia can affect physiological parameters. In each experiment, we strictly controlled the actual gas flow and anesthetic dose for the mice using anesthesia equipment, while also monitoring the mice’s heart and respiration rates and maintaining their body temperature to minimize the impact of anesthesia on the experimental results.

There are still some limitations of this study that need to be considered. Firstly, while this model accurately simulates the process of vascular stenosis and recanalization, it does not fully replicate the complex etiologies of human TIA, which may involve atherosclerosis, cardiogenic embolism, and other factors [1]. Secondly, neuroprotective agents play a crucial role in the treatment of ischemic stroke by protecting the neural tissue through mechanisms such as reducing apoptosis and antioxidation [53]. However, in our model, the use of isoflurane anesthesia, which itself has neuroprotective effects, may influence the assessment of the efficacy of neuroprotective agents. Thirdly, clinical studies have shown that TIA patients exhibit abnormal functional connectivity, including reduced connections in the somatomotor and visual networks [54,55]. In this study, thrombi were observed in the sensorimotor and visual cortexes after ischemia, which may contribute to the disrupted functional connectivity in these networks following TIA. However, our study lacked direct monitoring of the functional connectivity. Lastly, the long-term effects and translational applications of our model require further validation. While we observed some pathological changes in the acute phase, the research on long-term outcomes and functional recovery is insufficient.

In conclusion, by utilizing spatially targeted photothrombosis, which allowed for flexible control of the light spot’s shape and position, and combining it with real-time blood flow imaging feedback for precise control of the light dose, we constructed a dMCA-based photothrombotic TIA model consistent with the clinical definition of TIA. This method enabled the dynamic observation of the ischemic process and its impact on cerebral blood flow, including collateral circulation, during the acute phase of TIA. This approach provides a powerful tool for TIA studies.

## 4. Materials and Methods

### 4.1. Animals and Surgery

Male C57BL/6JNifdc mice, aged 10–16 weeks, were obtained from commercial vendors (Charles River, Beijing, China). Since estrogen has been shown to have neuroprotective effects that could alleviate brain ischemic injury [56], the experiment was conducted using male mice. The mice were acclimatized to a 12 h light/dark cycle with free access to food and water. All procedures involving animals were approved by the Hubei Provincial Animal Care and Use Committee and were in accordance with the experimental guidelines of the Animal Experimentation Ethics Committee of Huazhong University of Science and Technology, Wuhan, China.

**Figure 8:**
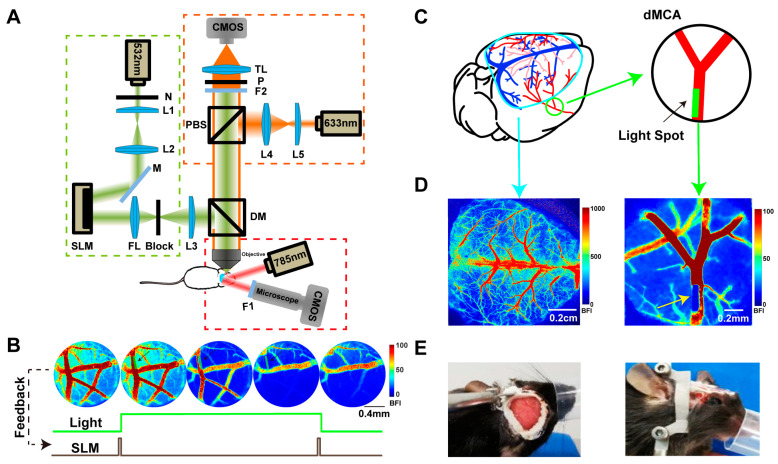
Animal model and system schematic for spatially targeted photochemistry with real-time flow imaging feedback. (**A**) Schematic of the experimental system. The green dashed box indicates flexible spatial targeting photothrombosis, orange dashed box indicates real-time blood flow imaging feedback, and the red dashed box indicates dorsal cerebral cortex laser speckle blood flow imaging (N: neutral density filter; L1–L5: lenses; M: mirror; SLM: spatial light modulators; FL: Fourier lens; DM: dichroic mirrors; PBS: polarization beam splitter; P: polarizer; TL: tube lens; F1, F2: filters). (**B**) The top row displays BFI images captured over time. Based on the feedback from the BFI images, the SLM loaded and removed the phase map (bottom brown trace), thereby controlling the switching of the light illumination (middle green trace). (**C**) Diagram of the relative position of the dMCA (green circle) to the dCC (outlined in light blue) window on the left and a magnified view of the dMCA window on the right (red: MCA; green box: light spot). (**D**) Typical BFI images of the dCC (left) and dMCA (right). The area indicated by the yellow arrow shows the light spot. (**E**) Mouse model and fixation frame. Left: dCC window; right: dMCA window.

The animal model was primarily divided into two main parts—the dCC window and the dMCA window. The relative positions of the two windows are shown in the Figure 8C. Throughout the surgical and experimental procedures, mice were anesthetized with isoflurane (2% for induction and 1 to 1.5% for maintenance) in medical O2. The body temperature of the mouse was maintained at 36.8–37 °C with a homeothermic pad controlled by a rectal thermometer (RWD, Shenzhen, China).

The method for creating the dCC window was as follows (left panels in Figure 8C–E). Following induction of anesthesia, each animal was placed on a stereotaxic frame in a prone position and its head was stabilized with ear bars. A lubricant ointment was applied to the eyes to prevent drying. After exposing the dorsal skull, the bone above the cortex over both hemispheres measuring approximately 8 mm in diameter (3 mm anterior and 4 mm lateral to bregma) was thinned by a dental drill until it was translucent, with the pial vessels clearly visible through the wetted bone [57]. The dCC window was surrounded by Superbond C&B dental cement (Sun Medical Co., Ltd., Moriyama, Japan). The clear version of C&B-Metabond dental cement (Parkell, Edgewood, NY, USA) was put on the thinned region and a cover glass was used to cap the cement [58].

The dMCA window was designed to induce photochemical ischemia (right panels in Figure 8C–E). To confirm whether the actual light position was the same as the selected position, we captured a picture without green light filtering before the ischemia. The position of the green laser irradiation in dMCA is shown as dark blue in the blood flow map and the dark blue square pointed out by the yellow arrow in Figure 8D is the actual position of the green laser irradiation. The surgical model referred to the commonly used electrocoagulation ischemia model [59]. Each anesthetized mouse was placed in a lateral position on the surgery table and the skin between the lateral part of the orbit and the external auditory canal was removed. The temporal muscle underlying the skin was partially dissected and pushed aside to expose the skull. After resecting the muscle, the cortical arteries were viewed through the partially translucent mouse skull. A round window with a diameter of 2 mm was created using a dental drill to expose the dMCA. Then, a circular glass coverslip measuring 2 mm in diameter, which had been fixed to a metal ring with an inner diameter of 1.5 mm and an outer diameter of 2.5 mm using UV glue in advance, was placed over the exposed dura and sealed with superglue along the edges. Additionally, a homemade fixation piece was secured with dental cement next to the imaging window (Figure 8E). The surgery was completed and the mouse was returned to a cage with a heating pad at the bottom. To relieve inflammation and pain, dexamethasone (2 mg/kg) and carprofen (2.5 mg/kg) were injected subcutaneously after surgery.

### 4.2. Imaging System

The imaging system was composed of three parts (Figure 8), namely flexible spatial targeting photothrombosis (green dashed box), real-time blood flow imaging feedback (orange dashed box), and dorsal cerebral cortex laser speckle blood flow imaging (red dashed box).

In order to induce flexible spatial targeting photothrombosis, a green laser beam (532 nm, LR-GSP-532/300 mW; Changchun Laser Technology, Changchun, China) was sequentially passed through a continuous neutral-density filter (N in Figure 8A; NDC-50C-2, Thorlabs, Newton, NJ, USA) and a collimated beam expansion lens set and then directed on the surface of a spatial light modulator (SLM, P1920-0523 HDMI; Meadowlark Optics, Longmont, CO, USA). The beam, which was modulated by the SLM, passed through a Fourier lens (FL in Figure 8A, f = 200 mm; LA1708, Thorlabs, Newton, NJ, USA) and a block before being directed onto the dMCA window using dichroic mirrors (DM in Figure 8A; DMLP638R, Thorlabs, Newton, NJ, USA). The block was placed on the back focal plane to remove the zero-order light spot.

An extended red laser beam (He-Ne laser, 632.8 nm, 15 mW; Thorlabs, Newton, NJ, USA) designed for LSCI illuminated the dMCA window through a polarization beam splitter (PBS in Figure 8A, PBS251, Thorlabs, Newton, NJ, USA) and an objective lens (XLFLUOR4X/340, Olympus, Tokyo, Japan). The scattered light was then captured by a CMOS camera (acA2040-120um, Basler, Ahrensburg, Germany; the other two cameras were the same model) equipped with a long-pass filter (F2 in Figure 8A; BLP01-594R-25, Semrock, Rochester, NY, USA) for real-time blood flow imaging feedback of the dMCA.

The self-designed Labview software (Labview 2017, National Instruments, Austin, TX, USA) was used to control the integrated system. The position and shape to be irradiated were manually selected on the real-time laser speckle imager, and this information was used to obtain a corresponding binary map. After loading the phase map calculated with the weighted Gerchberg–Saxton (GSW) algorithm in MATLAB (2017a, MathWorks, Natick, MA, USA) on the SLM, the required diffraction spatial pattern can be formed behind the Fourier lens. The diffraction pattern is then imaged on the focal plane of the objective lens through a microscopic imaging system, achieving spatial light stimulation of any position, shape, and size [24]. It was possible to induce photochemical ischemia (PI) over a wide range or a single large vessel and to target single or multiple microvessels for spatially targeted photochemical ischemia. Additionally, the real-time blood flow imaging feedback allowed for the observation of blood flow changes in the dMCA and the precise control of the laser irradiation duration during PI to induce varying degrees of ischemia.

For LSCI of the dCC window, an uncollimated, unfocused near-infrared laser beam (785 nm, laser diode; Thorlabs, Newton, NJ, USA) was used to irradiate the dCC window through a microscope (Z16 APO, Leica, Wetzlar, Germany) and a bandpass filter (F1 in Figure 8A; FF01-785/62-25, Semrock, Rochester, NY, USA). The filtered light was then captured by a CMOS camera.

### 4.3. Photochemical Ischemia

In the experiment, the mice were immobilized on a homemade fixator while lying on their right side (Figure 8E). Rose Bengal dye (Sigma-Aldrich, St. Louis, MO, USA, 30 mg/kg) was injected through the tail vein two minutes before imaging (the control group was injected with an equal amount of saline). The green laser was modulated by the SLM into a square spot measuring 50 × 250 μm size, with a width of about half the diameter of the dMCA (green square indicated by the black arrow in Figure 8B). The laser intensity under the objective lens was set at 3 mW when the patterned illumination was applied and the laser power density was 139 mW/mm2. LSCI was used to provide real-time blood flow feedback during the occurrence of distal middle artery ischemia. As shown in Figure 8B, the SLM loaded the phase map and started the light illumination, causing the dMCA to gradually narrow. Once the real-time blood flow imaging showed the complete occlusion of the dMCA, the feedback control stopped the SLM from loading the phase map, thereby ceasing the illumination. When the green laser illumination ceased, the LSCI of the dMCA continued without interruption. Simultaneously, the LSCI of the cerebral cortex was conducted with an initial acquisition rate of 10 Hz, which was adjusted to 2 Hz or 1 Hz (secondary PI) after 400 s or 500 s. The exposure time was set at 10 ms. By monitoring the blood flow on both sides of the dMCA and the cerebral cortex, it was possible to dynamically observe the temporal and spatial dynamics of the cortical blood flow in real time throughout the entire sequence, starting from stenosis to embolism and then from embolism to recanalization. The control group’s blood flow remained at baseline without any changes throughout the experiment (Figure S1).

### 4.4. TTC Staining

The infarct volume was measured using TTC, a standard method for the fast and reliable delineation of cerebral ischemic injury [60]. Twenty-four hours after the onset of transient ischemia, the mice were euthanized and the intact brains were rapidly removed and washed in PBS (pH 7.4). Brain tissue from an area 4 mm anterior and 5 mm posterior to the bregma was cut into serial 1 mm thick slices using a mouse brain slicer. The sliced brain tissue samples were stained with 2% TTC (Sigma-Aldrich, St. Louis, MO, USA) for 30 min at 37 °C in the dark, followed by overnight immersion in 4% paraformaldehyde (PFA) in 0.1 M PBS at pH 7.4 and 4 °C [61]. Images of TTC-stained brain sections were obtained using a digital camera.

### 4.5. Antibodies and Fluorescent Tracers

To track the locations of thrombi formed by PI in the mouse brain, we labeled platelets with CD41 in vivo. Additionally, endothelial cells were labeled with CD31. Antibodies were added to sterile 0.9% NaCl solution to a final infusion volume of 100 μL. The used antibodies and antibody concentrations were as follows: platelets—rat anti-mouse CD41-Alexa Fluor 647, 7 μL of 500 μg/mL (cat.#133933, clone MWReg30, Biolegend, San Diego, CA, USA); sinusoidal endothelial cells—rat anti-mouse CD31-PE, 10 μL of 200 μg/mL (cat.#12-0311-83, clone 390, eBioscience, San Diego, CA, USA) [46,62]. The mixture was infused into the tail vein directly 2 h before PI using a 1 mL insulin syringe. At 10 min, 4 h, or 24 h after PI, the mice were not subjected to cardiac perfusion but were directly executed for brain removal in order not to affect the location of the thrombus. The intact brains were carefully removed and post-fixed in 4% PFA for at least 24 h. The brain tissues were cut into 100-µm-thick coronal sections using a vibrating microtome (CM 1950, Leica, Wetzlar, Germany) and mounted onto glass slides for subsequent imaging. After applying a coverslip, the sections were analyzed with an inverted confocal microscope (LSM 710, Zeiss, Oberkochen Germany). The thrombi were manually counted in representative and different fields at various times using Image J software (Version 1.53c, NIH, Bethesda, MD, USA).

### 4.6. Cylinder Test

The cylinder test provides a way to evaluate a rodent’s spontaneous forelimb use and has been used in a number of motor system injury models of stroke [63]. The apparatus for this test includes an 8-cm-wide, 25-cm-high transparent cylinder and two mirrors. Two mirrors were attached to the cylinder, positioned vertically and at a 90 degree angle to each other, facilitating the recording of the back side of the cylinder. The mice were put into the cylinder 3 days before the surgery, and those who were overly anxious, jumped out of the cylinder, or touched the walls less than five times within 5 min were excluded. One day before the ischemia experiment, the mice were placed in the cylinder again and the number of times they touched the wall within 5 min was counted [64]. The number of times the left forelimb touched the wall was recorded as L and by the right as R. The left-to-right ratio N=R/L was calculated. Afterward, the same experiments were carried out again at 2 h, 6 h, and 24 h post-ischemia to compare changes in the value of N.

### 4.7. Data Analysis

We used a spatiotemporal laser speckle contrast analysis in which the speckle contrast was calculated directly from a 7 × 7 × 5 pixel cube [65,66,67]. The BFI was obtained using a laser speckle contrast analysis. The Ostu algorithm was used to binarize the blood flow image and calculate the dMCA diameter (D). The blood flow (BF) is represented by the product of the BFI and the cross-sectional area of the dMCA (see Equation (1)):

(1) $$BF=\frac{\pi D^{2}}{4}\times BFI$$

To demonstrate the longitudinal changes in blood flow and vessel diameter during the TIA, we selected five adjacent ROIs in the dMCA of a representative mouse for our analysis. We obtained the mean and standard deviation curves of the relative changes in blood flow and diameter over time. Additionally, for the analysis of the collateral circulation, we selected the most significantly developed collateral vessel in the cortex and analyzed its BFI’s relative changes over time in the same manner. The relationship between changes in the dMCA and BFI in the dorsal cortical was reflected by the Pearson correlation coefficients (see Equation (2)):

(2) $$R=\frac{cov\left(X,Y\right)}{\left(\sigma x\times \sigma y\right)}$$

where $cov\left(X,Y\right)$ represents the covariances of *X* and *Y* and *σx* and *σy* are the standard deviations of *X* and *Y*, respectively. Here, *X* denotes the relative changes over time in diameter or blood flow at the dMCA light spot, while *Y* signifies the relative changes over time in blood flow across each pixel of the dorsal cortex. All calculations were carried out using MATLAB (2017a, MathWorks, Natick, MA, USA).

### 4.8. Statistical Analysis

All data are expressed as means ± SD and the data analysis was carried out using GraphPad Prism (GraphPad Prism 9.0.0, San Diego, CA, USA). The statistical significance was determined using unpaired *t*-tests or paired *t*-tests for comparisons between two groups and a one-way analysis of variance (ANOVA) followed by Tukey’s post hoc test for comparisons among multiple groups. A probability value of less than 0.05 was considered to represent statistical significance.

## Data Availability

The data presented in this study are available upon request from the corresponding author.

## Supplementary Materials

The following supporting information can be downloaded at: https://www.mdpi.com/article/10.3390/ijms25147557/s1.

## References

[1] Easton J.D., Saver J.L., Albers G.W., Alberts M.J., Chaturvedi S., Feldmann E., Hatsukami T.S., Higashida R.T., Johnston S.C., Kidwell C.S. (2009). Definition and Evaluation of Transient Ischemic Attack: A Scientific Statement for Healthcare Professionals from the American Heart association/American Stroke Association Stroke Council; Council on Cardiovascular Surgery and Anesthesia; Council on Cardio. Stroke.

[2] Rothwell P.M., Warlow C.P. (2005). Timing of TIAs Preceding Stroke: Time Window for Prevention Is Very Short. Neurology.

[3] Kleindorfer D.O., Towfighi A., Chaturvedi S., Cockroft K.M., Gutierrez J., Lombardi-Hill D., Kamel H., Kernan W.N., Kittner S.J., Leira E.C. (2021). 2021 Guideline for the Prevention of Stroke in Patients with Stroke and Transient Ischemic Attack: A Guideline from the American Heart Association/American Stroke Association. Stroke.

[4] Kalyuzhnaya Y.N., Khaitin A.M., Demyanenko S.V. (2023). Modeling Transient Ischemic Attack via Photothrombosis. Biophys. Rev..

[5] Durukan Tolvanen A., Tatlisumak E., Pedrono E., Abo-Ramadan U., Tatlisumak T. (2017). TIA Model Is Attainable in Wistar Rats by Intraluminal Occlusion of the MCA for 10 Min or Shorter. Brain Res..

[6] Pedrono E., Durukan A., Strbian D., Marinkovic I., Shekhar S., Pitkonen M., Abo-Ramadan U., Tatlisumak T. (2010). An Optimized Mouse Model for Transient Ischemic Attack. J. Neuropathol. Exp. Neurol..

[7] Wang J., Li Y., Yu H., Li G., Bai S., Chen S., Zhang P., Tang Z. (2021). Dl-3-N-Butylphthalide Promotes Angiogenesis in an Optimized Model of Transient Ischemic Attack in C57BL/6 Mice. Front. Pharmacol..

[8] Berthet C., Lei H., Gruetter R., Hirt L. (2011). Early Predictive Biomarkers for Lesion after Transient Cerebral Ischemia. Stroke.

[9] Ejaz S., Emmrich J.V., Sawiak S.J., Williamson D.J., Baron J.C. (2015). Cortical Selective Neuronal Loss, Impaired Behavior, and Normal Magnetic Resonance Imaging in a New Rat Model of True Transient Ischemic Attacks. Stroke.

[10] Knapp L., Gellért L., Herédi J., Kocsis K., Oláh G., Fuzik J., Kis Z., Vécsei L., Toldi J., Farkas T. (2014). A Simple Novel Technique to Induce Short-Lasting Local Brain Ischaemia in the Rat. Neuropathol. Appl. Neurobiol..

[11] Barthels D., Das H. (2020). Current Advances in Ischemic Stroke Research and Therapies. Biochim. Biophys. Acta—Mol. Basis Dis..

[12] Schroeter M., Jander S., Stoll G. (2002). Non-Invasive Induction of Focal Cerebral Ischemia in Mice by Photothrombosis of Cortical Microvessels: Characterization of Inflammatory Responses. J. Neurosci. Methods.

[13] Sun Y.-Y., Kuo Y.-M., Chen H.-R., Short-Miller J.C., Smucker M.R., Kuan C.-Y. (2020). A Murine Photothrombotic Stroke Model with an Increased Fibrin Content and Improved Responses to tPA-Lytic Treatment. Blood Adv..

[14] Uzdensky A.B. (2018). Photothrombotic Stroke as a Model of Ischemic Stroke. Transl. Stroke Res..

[15] Tuor U.I., Deng Q., Rushforth D., Foniok T., Qiao M. (2016). Model of Minor Stroke with Mild Peri-Infarct Ischemic Injury. J. Neurosci. Methods.

[16] Mozaffarian D., Benjamin E.J., Go A.S., Arnett D.K., Blaha M.J., Cushman M., De Ferranti S., Després J.P., Fullerton H.J., Howard V.J. (2015). Heart Disease and Stroke Statistics-2015 Update: A Report from the American Heart Association. Circulation.

[17] Taylor Z., Shih A. (2013). Targeted Occlusion of Individual Pial Vessels of Mouse Cortex. Bio-Protocol.

[18] Delafontaine-Martel P., Zhang C., Lu X., Damseh R., Lesage F., Marchand P.J. (2023). Targeted Capillary Photothrombosis via Multiphoton Excitation of Rose Bengal. J. Cereb. Blood Flow Metab..

[19] Liu Z., He B., Wang X., Peng J., Sun Q., Luo C. (2023). Deep Cortical Microinfarction Induced by Femtosecond Laser in Mice: Long-Term Secondary Pathological Changes in Corresponding Superficial Cortex. Neurosci. Lett..

[20] Yao H., Nabika T. (2011). Characterizing Photothrombotic Distal Middle Cerebral Artery Occlusion and YAG Laser-Induced Reperfusion Model in the Izumo Strain of Spontaneously Hypertensive Rats. Cell. Mol. Neurobiol..

[21] Qian C., Li P.-C., Jiao Y., Yao H.-H., Chen Y.-C., Yang J., Ding J., Yang X.-Y., Teng G.-J. (2016). Precise Characterization of the Penumbra Revealed by MRI: A Modified Photothrombotic Stroke Model Study. PLoS ONE.

[22] Vaz P.G., Humeau-Heurtier A., Figueiras E., Correia C., Cardoso J. (2016). Laser Speckle Imaging to Monitor Microvascular Blood Flow: A Review. IEEE Rev. Biomed. Eng..

[23] Wang Z., Luo W., Zhou F., Li P., Luo Q. (2012). Dynamic Change of Collateral Flow Varying with Distribution of Regional Blood Flow in Acute Ischemic Rat Cortex. J. Biomed. Opt..

[24] Wen D., Li Y., Zhu X., Chen M., Lu J., Li P. (2018). Selective Photoactivation of Neural Activity Combined with Laser Speckle Imaging of Cerebral Blood Flow. Opt. Lett..

[25] Leigh R., Knutsson L., Zhou J., van Zijl P.C.M. (2018). Imaging the Physiological Evolution of the Ischemic Penumbra in Acute Ischemic Stroke. J. Cereb. Blood Flow Metab..

[26] Ermine C.M., Bivard A., Parsons M.W., Baron J.C. (2021). The Ischemic Penumbra: From Concept to Reality. Int. J. Stroke.

[27] Zhao H.T., Tuohy M.C., Chow D., Kozberg M.G., Kim S.H., Shaik M.A., Hillman E.M.C. (2021). Neurovascular Dynamics of Repeated Cortical Spreading Depolarizations after Acute Brain Injury. Cell Rep..

[28] Lee V.M., Burdett N.G., Carpenter T.A., Hall L.D., Pambakian P.S., Patel S., Wood N.I., James M.F. (1996). Evolution of Photochemically Induced Focal Cerebral Ischemia in the Rat: Magnetic Resonance Imaging and Histology. Stroke.

[29] Durukan A., Tatlisumak T. (2008). Chapter 3 Animal Models of Ischemic Stroke. Handbook of Clinical Neurology.

[30] Purroy F., Jimenez Caballero P.E., Gorospe A., Torres M.J., Alvarez-Sabin J., Santamarina E., Martinez-Sanchez P., Canovas D., Freijo M.J., Egido J.A. (2013). Recurrent Transient Ischaemic Attack and Early Risk of Stroke: Data from the PROMAPA Study. J. Neurol. Neurosurg. Psychiatry.

[31] Coutts S.B., Modi J., Patel S.K., Demchuk A.M., Goyal M., Hill M.D. (2012). CT/CT Angiography and MRI Findings Predict Recurrent Stroke After Transient Ischemic Attack and Minor Stroke. Stroke.

[32] Wang J., Zhang P., Tang Z. (2020). Animal Models of Transient Ischemic Attack: A Review. Acta Neurol. Belg..

[33] Conti E., Carlini N., Piccardi B., Allegra Mascaro A.L., Pavone F.S. (2023). Photothrombotic Middle Cerebral Artery Occlusion in Mice: A Novel Model of Ischemic Stroke. eNeuro.

[34] Yoo H.J., Ham J., Duc N.T., Lee B. (2021). Quantification of Stroke Lesion Volume Using Epidural EEG in a Cerebral Ischaemic Rat Model. Sci. Rep..

[35] Zhu L., Wang M., Fu P., Liu Y., Zhang H., Roe A.W., Xi W. (2023). Precision 1070 Nm Ultrafast Laser-Induced Photothrombosis of Depth-Targeted Vessels In Vivo. Small Methods.

[36] Fukuda M., Matsumura T., Suda T., Hirase H. (2022). Depth-Targeted Intracortical Microstroke by Two-Photon Photothrombosis in Rodent Brain. Neurophotonics.

[37] Jeng J.-S., Hsieh F.-I., Yeh H.-L., Chen W.-H., Chiu H.-C., Tang S.-C., Liu C.-H., Lin H.-J., Hsu S.-P., Lo Y.-K. (2017). Impact of MCA Stenosis on the Early Outcome in Acute Ischemic Stroke Patients. PLoS ONE.

[38] Dreier J.P. (2011). The Role of Spreading Depression, Spreading Depolarization and Spreading Ischemia in Neurological Disease. Nat. Med..

[39] Lauritzen M., Dreier J.P., Fabricius M., Hartings J.A., Graf R., Strong A.J. (2011). Clinical Relevance of Cortical Spreading Depression in Neurological Disorders: Migraine, Malignant Stroke, Subarachnoid and Intracranial Hemorrhage, and Traumatic Brain Injury. J. Cereb. Blood Flow Metab..

[40] Jurcau A., Simion A. (2021). Neuroinflammation in Cerebral Ischemia and Ischemia/Reperfusion Injuries: From Pathophysiology to Therapeutic Strategies. Int. J. Mol. Sci..

[41] Arsava E.M., Gurer G., Gursoy-Ozdemir Y., Karatas H., Dalkara T. (2009). A New Model of Transient Focal Cerebral Ischemia for Inducing Selective Neuronal Necrosis. Brain Res. Bull..

[42] Walz E.T., Brink T., Slivka A. (1997). Pattern and Frequency of Recurrent Transient Ischemic Attacks. J. Stroke Cerebrovasc. Dis..

[43] Wang L., Chaudhari K., Winters A., Sun Y., Berry R., Tang C., Yang S.-H., Liu R. (2023). Recurrent Transient Ischemic Attack Induces Neural Cytoskeleton Modification and Gliosis in an Experimental Model. Transl. Stroke Res..

[44] Tuor U.I., Zhao Z., Barber P.A., Qiao M. (2016). Recurrent Mild Cerebral Ischemia: Enhanced Brain Injury Following Acute Compared to Subacute Recurrence in the Rat. BMC Neurosci..

[45] Tomaiuolo M., Brass L.F., Stalker T.J. (2017). Regulation of Platelet Activation and Coagulation and Its Role in Vascular Injury and Arterial Thrombosis. Interv. Cardiol. Clin..

[46] Stalker T.J., Traxler E.A., Wu J., Wannemacher K.M., Cermignano S.L., Voronov R., Diamond S.L., Brass L.F. (2013). Hierarchical Organization in the Hemostatic Response and Its Relationship to the Platelet-Signaling Network. Blood.

[47] Lin X., Fan Y., Zhang F., Lin Y. (2022). Cerebral Microinfarct Is Emergency Consequence of Alzheimer’s Disease: A New Insight into Development of Neurodegenerative Diseases. Int. J. Biol. Sci..

[48] Wang M., Iliff J.J., Liao Y., Chen M.J., Shinseki M.S., Venkataraman A., Cheung J., Wang W., Nedergaard M. (2012). Cognitive Deficits and Delayed Neuronal Loss in a Mouse Model of Multiple Microinfarcts. J. Neurosci..

[49] Tsai M.J., Kuo Y.M., Tsai Y.H. (2014). Transient Ischemic Attack Induced by Melted Solid Lipid Microparticles Protects Rat Brains from Permanent Focal Ischemia. Neuroscience.

[50] Culp W.C., Woods S.D., Brown A.T., Lowery J.D., Hennings L.J., Skinner R.D., Borrelli M.J., Roberson P.K. (2013). Three Variations in Rabbit Angiographic Stroke Models. J. Neurosci. Methods.

[51] Xiong B., Li A., Lou Y., Chen S., Long B., Peng J., Yang Z., Xu T., Yang X., Li X. (2017). Precise Cerebral Vascular Atlas in Stereotaxic Coordinates of Whole Mouse Brain. Front. Neuroanat..

[52] Dickinson R., Peterson B.K., Banks P., Simillis C., Martin J.C.S., Valenzuela C.A., Maze M., Franks N.P. (2007). Competitive Inhibition at the Glycine Site of the N-Methyl-D-Aspartate Receptor by the Anesthetics Xenon and Isoflurane: Evidence from Molecular Modeling and Electrophysiology. Anesthesiology.

[53] Haupt M., Gerner S.T., Bähr M., Doeppner T.R. (2023). Neuroprotective Strategies for Ischemic Stroke—Future Perspectives. Int. J. Mol. Sci..

[54] Li R., Wang S., Zhu L., Guo J., Zeng L., Gong Q., He L., Chen H. (2013). Aberrant Functional Connectivity of Resting State Networks in Transient Ischemic Attack. PLoS ONE.

[55] Zhu T., Li L., Song Y., Han Y., Zhou C., Zhou D., Zhang F., Xue Q., Liu J., Zhao L. (2019). Altered Functional Connectivity within Default Mode Network in Patients with Transient Ischemic Attack: A Resting-State Functional Magnetic Resonance Imaging Study. Cerebrovasc. Dis..

[56] Inagaki T., Etgen A.M. (2013). Neuroprotective Action of Acute Estrogens: Animal Models of Brain Ischemia and Clinical Implications. Steroids.

[57] Drew P.J., Shih A.Y., Driscoll J.D., Knutsen P.M., Blinder P., Davalos D., Akassoglou K., Tsai P.S., Kleinfeld D. (2010). Chronic Optical Access through a Polished and Reinforced Thinned Skull. Nat. Methods.

[58] Silasi G., Xiao D., Vanni M.P., Chen A.C.N., Murphy T.H. (2016). Intact Skull Chronic Windows for Mesoscopic Wide-Field Imaging in Awake Mice. J. Neurosci. Methods.

[59] Llovera G., Roth S., Plesnila N., Veltkamp R., Liesz A. (2014). Modeling Stroke in Mice: Permanent Coagulation of the Distal Middle Cerebral Artery. J. Vis. Exp..

[60] Choi W.J., Li Y., Wang R.K. (2019). Monitoring Acute Stroke Progression: Multi-Parametric OCT Imaging of Cortical Perfusion, Flow, and Tissue Scattering in a Mouse Model of Permanent Focal Ischemia. IEEE Trans. Med. Imaging.

[61] Liao L.-D., Bandla A., Ling J.M., Liu Y.-H., Kuo L.-W., Chen Y.-Y., King N.K., Lai H.-Y., Lin Y.-R., Thakor N.V. (2014). Improving Neurovascular Outcomes with Bilateral Forepaw Stimulation in a Rat Photothrombotic Ischemic Stroke Model. Neurophotonics.

[62] van Golen R.F., Stevens K.M., Colarusso P., Jaeschke H., Heger M. (2015). Platelet Aggregation but Not Activation and Degranulation during the Acute Post-Ischemic Reperfusion Phase in Livers with No Underlying Disease. J. Clin. Transl. Res..

[63] Balkaya M.G., Trueman R.C., Boltze J., Corbett D., Jolkkonen J. (2018). Behavioral Outcome Measures to Improve Experimental Stroke Research. Behav. Brain Res..

[64] Schaar K.L., Brenneman M.M., Savitz S.I. (2010). Functional Assessments in the Rodent Stroke Model. Exp. Transl. Stroke Med..

[65] Duncan D.D., Kirkpatrick S.J. (2008). Spatio-Temporal Algorithms for Processing Laser Speckle Imaging Data. Proceedings of the Optics in Tissue Engineering and Regenerative Medicine II.

[66] Qiu J. (2010). Spatiotemporal Laser Speckle Contrast Analysis for Blood Flow Imaging with Maximized Speckle Contrast. J. Biomed. Opt..

[67] Briers D., Duncan D.D., Hirst E., Kirkpatrick S.J., Larsson M., Steenbergen W., Stromberg T., Thompson O.B. (2013). Laser Speckle Contrast Imaging: Theoretical and Practical Limitations. J. Biomed. Opt..

