# Supplementary material for: Constructing a Transient Ischemia Attack Model Utilizing Flexible Spatial Targeting Photothrombosis with Real-Time Blood Flow Imaging Feedback
Source: Int J Mol Sci. 2024 Jul 10;25(14):7557. doi: 10.3390/ijms25147557 (PMC11277306; doi:10.3390/ijms25147557)
Supplement: Supplementary file 1 [file ijms-25-07557-s001.zip › ijms-3067857-supplementary.pdf]

Supplementary Materials

Saline control group: When all other experimental conditions were the same and rose bengal was replaced with an equivalent amount of saline, BFI remained at baseline without any changes throughout the experiment (as shown below).

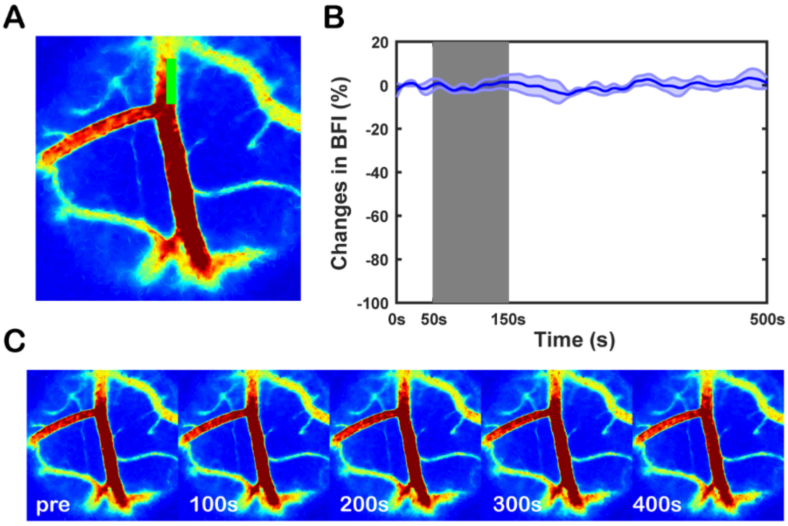

Figure S1. (**A**) Representative BFI images of baseline. The green box indicates the position of the light spot. (**B**) Relative change of BFI at the light spot, with the gray area indicating the duration of light. (**C**) Representative BFI images of the dMCA at various time points.
